# A primary hepatic lymphoma in a patient with Crohn's disease receiving thiopurine and anti-TNF therapy: a case report

**DOI:** 10.2144/fsoa-2023-0082

**Published:** 2023-08-28

**Authors:** Salma Merhaben, Shema Ayadi, Monia Tangour, Nadia Boujelbene, Rayfa Ghariani, Leila Mouelhi, Fathi Merhaben

**Affiliations:** 1 Gastroenterology Department, Charles Nicolle hospital, Tunis, 1006, Tunisia; 2 Histopathology Department, Salah Azaiez Institute, Tunis, 1007, Tunisia; 3 Radiology Department, El Amen private hospital, Nabeul, 8000, Tunisia; 4 Gastroenterology Department, El Amen private hospital, Nabeul, 8000, Tunisia

**Keywords:** anti-TNF therapy, Crohn's disease, diffuse large -B cell lymphoma, primary hepatic lymphoma, thiopurine

## Abstract

Primary hepatic lymphoma is a rare variant of non-Hodgkin's lymphoma with an incidence of 0.016% of all non-Hodgkin lymphomas. The most common histologic subtype is large diffuse B-cell lymphoma. Pathogenesis is not clearly established and undergoing immunosuppressive therapy has been proposed as a risk factor for primary hepatic lymphoma. We report an intriguing case study, featuring a 23-year-old male patient with Crohn's Disease who had been receiving a combination therapy of thiopurine and anti-TNF for 6 years and was diagnosed with primary hepatic diffuse large B-cell lymphoma.

## Plain language summary

Primary hepatic lymphoma is a rare type of cancer that mostly affects the liver and accounts for less than 1% of all lymphoma cases. The exact cause of primary hepatic lymphoma is unknown, but some evidence suggests that immunosuppressive therapy may increase the risk of developing this condition. We present a 23-year-old male patient who has a six-year history of Crohn's Disease, which is a long-lasting condition that causes inflammation. To manage his symptoms, he was taking two types of medications that weaken the immune system (thiopurine and anti-TNF medications). While being treated for Crohn's disease, he was also diagnosed with a rare type of primary hepatic lymphoma. This case raises intriguing questions about the relationship between immunosuppressive therapy and the development of primary hepatic lymphoma. It emphasizes the need for further research to better understand the underlying mechanisms and identify potential risk factors.

## Tweetable abstract

A rare discovery. We present a compelling case of a 23-year-old male with Crohn's Disease, on immunosuppressive therapy, diagnosed with primary hepatic diffuse large B-cell lymphoma. #RareDisease #LymphomaAwareness.

Thiopurine and anti-TNF therapies have become increasingly crucial in managing inflammatory bowel disease (IBD) in recent years.

However, one of the main concerns with these treatments is the increased risk of lymphoma. Lymph nodes and intestines are among the most commonly affected sites of lymphoma. Primary hepatic localization, however, is extremely rare, accounting for 0.016% of all non-Hodgkin lymphomas [1]. We present an interesting case report of primary hepatic diffuse large B-cells non-Hodgkin lymphoma in a 23-year-old male patient with Crohn's Disease (CD) who received a combination therapy of thiopurine and anti-TNF agents for 6 years.

## Case report

A 23-year-old male patient with no significant family history but a personal history of ileocolonic Crohn's disease with a stricturing phenotype presented to Charles Nicolle Hospital. Pretherapeutic investigations before introducing azathioprine therapy were negative, particularly for Epstein–Barr virus (EBV) infection markers. The patient was treated unsuccessfully with azathioprine monotherapy at the dosage of 2.3 mg/kg/day for 2 years, and then he had been treated with a combination therapy of thiopurine and Adalimumab for 6 years. Adalimumab was prescribed at the standard dose of 40 mg every 2 weeks for CD treatment. Clinical and biological remission was obtained but not endoscopic remission.

The patient presented in March 2023, with a 1-week history of abdominal pain. He did not mention any diarrhea or difficulties in gas or stool emission. There was no reported fever, night sweats, jaundice or weight loss. The patient denied any cigarette or alcohol consumption.

His physical examination revealed a good general health status (ECOG-PS 0), anicteric sclera, normal blood pressure, and a heart rate of 110 beats per minute. Tenderness of the right hypochondrium and epigastric region was noted. No peripheral lymph nodes or organomegaly were observed.

Hematological and biochemical investigations revealed inflammatory biologic syndrome with elevated CRP level of 197 mg/l, and thrombocytosis of 441,000/mm^3^ but with normal white blood cell count and hemoglobin level. Hepatic tests showed cholestasis:GGT and ALP measuring 4,5 and 1,5-fold the upper limit of normal (ULN). Total bilirubin level was normal. Elevated liver enzymes were noted with ASAT and ALAT levels equal to 1,6- and 1,7-fold ULN respectively.

**Figure 1.**
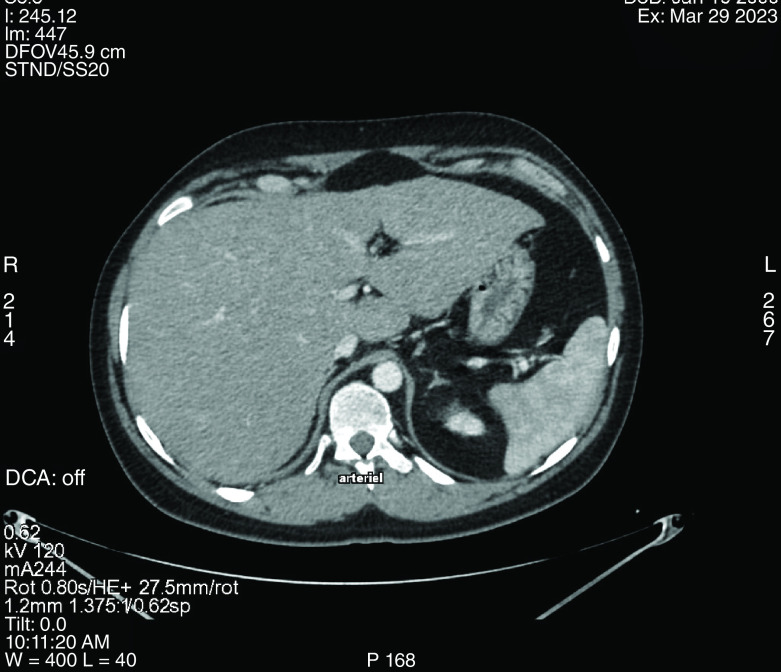
Abdominal CT scan showing a diffuse nodular hepatic infiltration.

Abdominal ultrasound and CT scan were performed excluding any intrahepatic or extrahepatic bile duct dilation or intraabdominal abscess. It revealed a heterogenous, nodular, and enlarged liver measuring 16 cm with globular contours. Sub-centimetric lymph nodes of the hepatic hilum were also noted on the CT scan (Figure 1).

In parallel, in order to explore liver enzymes elevation, the following tests were performed and revealed no abnormalities: EBV serology, blood cytomegalovirus antibody, hepatitis C virus (HCV) antibody, hepatitis B virus (HBV) antigen and human immunodeficiency virus (HIV) antibody. Quatiferon^®^ test excluded tuberculosis infection. There was no clinical, biological or morphologic sign of underlying cirrhosis or chronic advanced liver disease.

**Figure 2.**
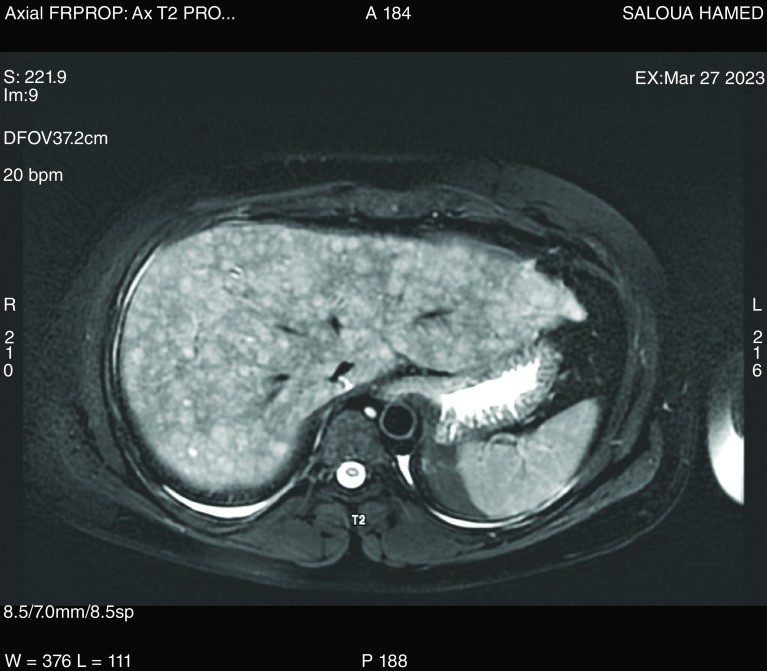
Liver MRI revealing a multiple nodular infiltration of the liver, with hyperintense signals on T2-weighted imaging.

A liver MRI was then conducted showing consistent results with a diffuse nodular hepatic infiltration. The nodules exhibited hypo-intense signals on T1-weighted imaging and hyperintense signals on T2-weighted imaging. Additionally, the MRI ruled out any associated cholangitis (Figure 2).

CEA and AFP levels were normal.

A CT-guided liver biopsy was conducted. Histological examination and immunohistochemistry confirmed the diagnosis of diffuse large B-cell liver lymphoma by revealing a proliferation of tumor cells with diffuse membranous CD20 staining.

**Figure 3.**
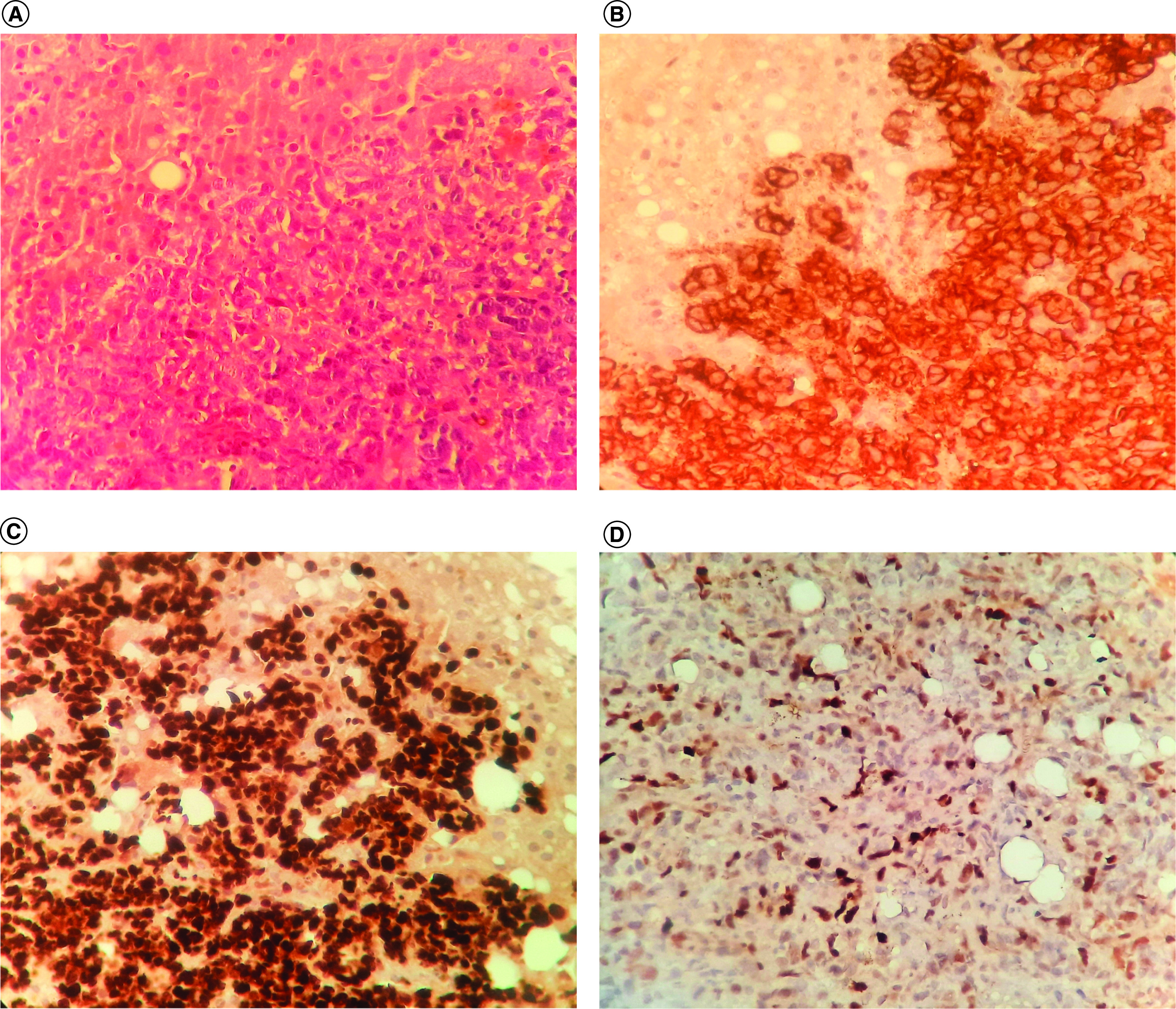
Histopathological and immunohistochemical features at liver biopsy. **(A)** Histopathological examination of liver biopsy with hematoxylin and eosin (H&E x 200) showing a small to medium lymphoid cell proliferation. **(B)** Lymphoid cells with diffuse membranous staining with CD20 (IHC x 200). **(C)** Extensive nuclear staining with Ki-67 reflecting a high proliferation rate (100%). (IHC x 200). **(D)** Negative staining for C-myc in the lymphoid population (only 10–20% of tumor cells).

Immunohistochemistry also showed an extensive nuclear staining with Ki-67 reflecting a high proliferation rate (100%). C-myc was negative, staining only 10–20% of tumor cells. (Figure 3A–D).

A staging assessment was conducted: cerebral and cervical-thoracic-abdominal-pelvic CT did not reveal any enlarged lymph nodes or organ infiltration, and bone marrow biopsy ruled out marrow infiltration. Additionally, upper and lower endoscopies with multiple systematic biopsies showed no malignant cell proliferation. There was high lactate dehydrogenase and beta 2 microglobulin levels.

A diagnosis of stage 1 according to Ann Arbor modified classification primary hepatic lymphoma (PHL) was made with an international prognostic index equal to 1.

Azathioprine and adalimumab treatment was discontinued, and the patient was referred to the hematology department for a combination of chemotherapy-rituximab therapy.

## Discussion

Immunosuppressive therapy and anti-tumor-necrosis factor agents (anti-TNF) are largely used in IBD treatment allowing an enhanced prognosis, especially in patients with severe inflammation. Endoscopic remission, a classic therapeutic goal in ulcerative colitis and most recently for Crohn's disease (CD), justified the increasing use of azathioprine and biotherapy in combination for IBD treatment [2].

However, concerns about potential complications associated with thiopurines and anti-TNF therapies have been raised. One of the main concerns with these treatments is the increased risk of opportunistic infections and malignancies, including lymphoma, especially when used in combination therapy.

Before determining whether a therapy raises the risk of lymphoma, it is important to remember that CD patients are at a higher risk for lymphoma, regardless of their exposure to thiopurines [3]. In fact, homozygote variants of the *NOD2* gene predisposes the carrier to Crohn's disease and potentially promotes the occurrence of hematological malignancies [3,4].

Otherwise, thiopurine use is linked with a well-established risk of lymphoma with a nearly fourfold higher incidence of lymphoma when compared with the general population [5].

However, the role of anti-TNF agents in inducing lymphoma remains unclear and controversial.

Initial reports showed a non-association between anti-TNF monotherapy and a higher risk of lymphoma [6–8]. More recently, a cohort study conducted by Lemaitre *et al.*, including 189–289 patients with a median follow-up of 6.7 years, reported a higher incidence of lymphoma among patients treated solely with anti-TNF therapy and no previous exposure to immunosuppressive therapy. The absolute risk difference in lymphoma incidence between exposed and unexposed patients was 0.15 per 1000 person-years [9]. This was attributed to the negative impact of anti-TNF treatment on the activation and function of NK cells, resulting in impaired IFN-g secretion, degranulation and decreased lysis of B lymphoma cell lines [10]. Furthermore, Lemaitre *et al.* found a similar risk of lymphoma in patients receiving thiopurine or anti-TNF in monotherapy and a higher risk for those under combination therapy [9].

In our case, using combo therapy with thiopurines and adalimumab can be criticized. In fact, only one randomized controlled trial (the DIAMOND trial) studied the use of combination therapy of adalimumab with thiopurine as compared with adalimumab monotherapy for the induction of clinical remission. In this trial, combination therapy was not superior to adalimumab monotherapy for inducing clinical remission [11].

The potential role of EBV status in the pathogenesis of lymphoproliferative disorders in IBD has been suggested with 46% of lymphoproliferative disorder cases reported EBV positive in the CESAME study [12]. This may be explained by Thiopurine's cytotoxicity for natural killer and cytotoxic T cells which have an important role in the control of EBV infection by eliminating EBV-infected B cells [13,14]. In our case, EBV serology was negative before starting the treatment with immunosuppressants and remained negative at diagnosis of primary hepatic lymphoma ruling out any possible induction by EBV infection.

In a recent French multicentric cohort, representing the largest published series of IBD patients diagnosed with lymphoma, lymphoma risk was about two-times greater in men than in women and was linked to immunosuppressive therapy duration. Higher risk was observed in young males under the age of 30, as well as in patients over the age of 50 [15]. However, it is important to note that the overall risk of developing lymphoma remains very low, estimated at 1 in 2000 patients per year for individuals under 50 years of age and 1 in 350 patients per year for those over 50 years of age [15]. In fact, over a period of 40 years, the French cohort study revealed only 52 cases of lymphoma in patients with IBD [15].

The most commonly observed lymphoma subtypes in patients with IBD were diffuse large B-cell lymphoma, Hodgkin lymphoma and follicular lymphoma [6].

Lymph nodes and intestines were the most described affected sites [14]. Primary hepatic localization, as in our current report, are extremely rare. Only one case of Hodgkin-like primary hepatic lymphoma was reported in the CESAME study [14].

In fact, PHL is a rare disease, accounting for 0.1% of all liver malignancies, 0.4% of extra-nodal non-Hodgkin lymphomas and 0.016% of all non-Hodgkin lymphomas [14,16].

To define the condition as PHL, either the liver must be the sole site of lymphoma occurrence or it must be significantly involved with minimal non-hepatic disease [18].

The largest study cohort, including 1372 patients with primary hepatic lymphoma, was published in 2021 and was based on a population analysis in USA [17].

In this study, male patients outnumbered female patients by approximately two-times. the median age at diagnosis was 65 years (range, 3–95 years). The majority of PHL cases (82.66%) were reported among white individuals.

PHL patients exhibited a range of nonspecific symptoms, such as fever, night sweats, abdominal pain and hepatomegaly.

PHL pathological features are similar to those of common liver tumors and can be classified into three types: uni-nodular, characterized by a single lesion >3 cm in diameter; multinodular, characterized by multiple lesions of varying size, usually <3 cm in diameter; diffuse or hepatomegaly, In this type, distinct lesions are absent, and the liver is characterized by diffuse involvement or enlargement. The diagnosis of PHL revealed that 670 patients (48.83%) were at an early stage (I and II), whereas 636 patients (51.36%) were diagnosed with an advanced stage (III and IV). Non-Hodgkin lymphoma (NHL) was found in 79.15% of PHL patients, while Hodgkin lymphoma accounted for only 2.41% of cases [17].

Among NHL subtypes, diffuse large B-cell lymphoma (DLBCL) was the most prevalent (78.82%), followed by T/NK-cell lymphoma (4.69%), marginal zone lymphoma (MZL; 4.51%), Burkitt's lymphoma (4.33%), and follicular lymphoma. Most patients did not undergo surgery or radiotherapy, with chemotherapy being administered to 65.16% of them.

Many etiologic factors are suspected to be implicated with primary hepatic lymphoma like Epstein–Barr virus (EBV) prior infection, hepatitis B and C (HBV, HCV), cirrhosis, and patients given immunosuppressive therapy [17]. To note, the underlying disease justifying immunosuppressive therapy was not mentioned in this cohort.

Patients with PHL had a median overall survival (OS) of 56.02 months.

Factors such as advanced age, unfavorable histologic subtypes, and advanced-stage disease are associated with poor prognosis in PHL patients [12,17], and treatment typically involves chemotherapy, radiation therapy, surgery, or a combination of these methods. In some cases, liver transplantation may be a viable option [17].

Treating IBD after lymphoma control is particularly challenging. Determining the appropriate treatment for a patient with an active IBD and a history of cancer presents its own difficulties. The 2015 ECCO recommendations on IBD and malignancies state that the risk of recurrence for lymphomas is low and that thiopurines should be avoided in such cases. Anti-TNF therapies, methotrexate and corticosteroids may be used with caution on a case-by-case basis, in a multidisciplinary fashion [3]. In our case, Adalimumab and azathioprine were stopped and chemotherapy-rituximab combination therapy is being conducted. In case of a CD flare-up during anti-tumor therapy, corticosteroids can be proposed. Methotrexate or anti-TNF therapies can be considered when corticodependence or resistance occurs. This decision would be best made in a multidisciplinary consultation meeting.

## Conclusion

To summarize, PHL is a very rare variant of non-Hodgkin's lymphoma. We present an exceptional case of a young male with CD, undergoing azathioprine and Adalimumab combination therapy and who was diagnosed with primary hepatic diffuse B-cell lymphoma.

Our case is noteworthy for two reasons: first, the rarity of lymphoma induced by immunosuppressive therapies, and second, the unique hepatic localization of the lymphoma.

Although cases of lymphoproliferative disorder due to treatment modalities used for IBD are rare, an increase in the risk of lymphoproliferative diseases should be considered in patients with IBD treated with immunomodulatory agents.

**Summary points**

- Thiopurine and anti-TNF therapies have become increasingly crucial in the management of inflammatory bowel disease (IBD) in recent years.
- One of the main concerns with these treatments is the increased risk of lymphoma.
- We present an exceptional case of a young male with Crohn's Disease (CD), undergoing azathioprine and Adalimumab combination therapy and who was diagnosed with primary hepatic lymphoma.
- A CT-guided liver biopsy was conducted. Histological examination and immunohistochemistry confirmed the diagnosis of diffuse large B-cell liver lymphoma.
- CD patients are at a higher risk for lymphoma, regardless of their exposure to thiopurines.
- Thiopurine use is linked with a well-established risk of lymphoma.
- The role of anti-TNF agents in inducing lymphoma remains unclear and controversial.
- Patients receiving a combination therapy of thiopurine and anti-TNF therapy are at a higher risk of developing a lymphoma than those receiving thiopurine or anti-TNF agent as a monotherapy.
- The role of Epstein–Barr virus (EBV) status in the pathogenesis of lymphoproliferative disorders in IBD is highly suggested.
- PHL is a rare disease, accounting for 0.1% of all liver malignancies, 0.4% of extra-nodal non-Hodgkin lymphomas, and 0.016% of all non-Hodgkin lymphomas.
- To define the condition as PHL, either the liver must be the sole site of lymphoma occurrence or it must be significantly involved with minimal non-hepatic disease.
- Many etiologic factors are suspected to be implicated with primary hepatic lymphoma like EBV prior infection, hepatitis B and C (HBV, HCV), cirrhosis, and patients given immunosuppressive therapy.
- Further investigation into PHL risk factors, pathogenesis, and optimal management strategies is needed.
